# Changes in Proteome and Protein Phosphorylation Reveal the Protective Roles of Exogenous Nitrogen in Alleviating Cadmium Toxicity in Poplar Plants

**DOI:** 10.3390/ijms21010278

**Published:** 2019-12-31

**Authors:** Jinliang Huang, Xiaolu Wu, Feifei Tian, Qi Chen, Pengrui Luo, Fan Zhang, Xueqin Wan, Yu Zhong, Qinglin Liu, Tiantian Lin

**Affiliations:** 1 College of Forestry, Sichuan Agricultural University, Chengdu 611130, China; B20162201@stu.sicau.edu.cn (J.H.); zhongyu315@163.com (Y.Z.); tlin@sicau.edu.cn (T.L.); 2 College of Landscape Architecture, Sichuan Agricultural University, Chengdu 611130, China; wuxiaolu@stu.sicau.edu.cn (X.W.); tianfeifei@stu.sicau.edu.cn (F.T.); chenqi@stu.sicau.edu.cn (Q.C.); luopengrui@stu.sicau.edu.cn (P.L.); 13854@sicau.edu.cn (Q.L.)

**Keywords:** poplar, cadmium stress, nitrogen, proteomics, phosphoproteome

## Abstract

Phytoremediation soil polluted by cadmium has drawn worldwide attention. However, how to improve the efficiency of plant remediation of cadmium contaminated soil remains unknown. Previous studies showed that nitrogen (N) significantly enhances cadmium uptake and accumulation in poplar plants. In order to explore the important role of nitrogen in plants’ responses to cadmium stress, this study investigates the poplar proteome and phosphoproteome difference between Cd stress and Cd + N treatment. In total, 6573 proteins were identified, and 5838 of them were quantified. With a fold-change threshold of > 1.3, and a *p*-value < 0.05, 375 and 108 proteins were up- and down-regulated by Cd stress when compared to the control, respectively. Compared to the Cd stress group, 42 and 89 proteins were up- and down-regulated by Cd + N treatment, respectively. Moreover, 522 and 127 proteins were up- and down-regulated by Cd + N treatment compared to the CK group. In addition, 1471 phosphosites in 721 proteins were identified. Based on a fold-change threshold of > 1.2, and a *p*-value < 0.05, the Cd stress up-regulated eight proteins containing eight phosphosites, and down-regulated 58 proteins containing 69 phosphosites, whereas N + Cd treatment up-regulated 86 proteins containing 95 phosphosites, and down-regulated 17 proteins containing 17 phosphosites, when compared to Cd stress alone. N + Cd treatment up-regulated 60 proteins containing 74 phosphosites and down-regulated 37 proteins containing 42 phosphosites, when compared to the control. Several putative responses to stress proteins, as well as transcriptional and translational regulation factors, were up-regulated by the addition of exogenous nitrogen following Cd stress. Especially, heat shock protein 70 (HSP70), 14-3-3 protein, peroxidase (POD), zinc finger protein (ZFP), ABC transporter protein, eukaryotic translation initiation factor (elF) and splicing factor 3 B subunit 1-like (SF3BI) were up-regulated by Cd + N treatment at both the proteome and the phosphoproteome levels. Combing the proteomic data and phosphoproteomics data, the mechanism by which exogenous nitrogen can alleviate cadmium toxicity in poplar plants was explained at the molecular level. The results of this study will establish the solid molecular foundation of the phytoremediation method to improve cadmium-contaminated soil.

## 1. Introduction

Heavy metal (HM) contamination has become a serious threat to the environment and human health [1]. Among heavy metals, cadmium (Cd), a widespread non-essential trace metal, is of great concern due to its widespread occurrence and high toxicity. Recently, Cd pollution has accelerated environmental deterioration. Notably, Cd contamination has widely degraded farmlands into the core polluted regions of the world [2,3]. Cd is easily taken up by plants through their root systems and can be accumulated through the food chain, which is a danger to animal and human health [4,5]. Cd has a negative effect on photosynthetic and nitrogen (N) metabolism in plants, leading to poor growth and a low biomass [6,7]. Additionally, Cd can stimulate the accumulation of reactive oxygen species (ROS), including the superoxide anion (O_2_^−^), hydroxyl radicals (OH) and hydrogen peroxide (H_2_O_2_), to disturb the redox balance in plants [8,9]. Therefore, the ability for ROS scavenging is vital for plant detoxification against Cd stress. According to previous research, the detoxification strategies of plants to Cd stress may include Cd sequestration, constraint, degradation, exclusion and inactivation by the exudation of organic ligands [10]. Sequestering Cd using phytochelatins (PCs), metallothioneins, organic acids, etc., and transporting Cd to the vacuole is confirmed to be an important pathway to detoxifying Cd [11].

These environmental and human health problems caused by heavy metals may be mitigated by many strategies. At present, many chemical, physical and biological techniques have been used to extract heavy metals from soils. However, among these techniques, phytoremediation has been proven to be effective and economical. Phytoremediation is a set of processes that uses plants to clean contamination in the environment [12,13,14]. Compared with traditional remediation technology, phytoremediation has been confirmed to be an optimal technique because it is a low-cost, sustainable and ecological method [15,16]. A number of different plants have an inherent ability to metabolize a variety of environmental pollutants [13,17]. Woody species, such as poplar and willow, have been used successfully for phytoremediation because of their characters of rapid growth, high-biomass, extensive root systems and amenability to transformation [18,19,20]. Additionally, using tall poplar and willow to remediate the contaminated environment is a functional approach that not only remediates the contaminated environment but also creates a landscape.

However, there are also several limitations for phytoremediation. Firstly, phytoremediation plants are often unique ecotypes and their specific habitations are limited [21,22]. Secondly, the ability to accumulate Cd varies significantly between species [23]. Thirdly, there is a lack of high-biomass Cd hyperaccumulators [24]. Fourth, phytoremediation is limited to shallow soils, streams and ground water. Fifth, high concentrations of hazardous materials can be toxic to plants. Therefore, how to improve the efficiency of phytoremediation is worthy of further study. Some documents have reported that nitrogen (N) can enhance the absorption of plants to Cd [25,26]. Li et al. [27] revealed that the addition of appropriate nitrogen to the nutrient solution could promote root system development and enhance the accumulation of Cd in *Sedum alfredii* Hance. Cd toxicity was mitigated by N deposition [11]. In leaves of *Pentas lanceolate*, N fertilization can promote the recovery of chlorophyll to alleviate the damages caused by Cd stress [28]. This study showed that excessive nitrates can enhance a plant’s uptake of Cd by up-regulating *OsIRT1* expression in rice [29]. Our previous study revealed that addition N can weaken the damage caused by Cd stress [30,31]. In a word, the existing documents suggest that nitrogen application can effectively alleviate the harm of cadmium to plants when N supply is optimal. However, the mechanisms behind heavy metal detoxification in plants remain unclear.

Proteins are very important molecules in cells and are involved in virtually all cell functions, such as resistance to biological and abiotic stresses. Differential proteomic analysis is achieving great success as a reliable and reproducible high-through put approach to study the molecular mechanisms of plant responses to heavy metals [32,33]. Additionally, protein phosphorylation is one of the most widespread post-translational modifications, as well as the key factor in controlling signal transduction, and phosphorylation may regulate heavy metal stress responses [34]. However, at present, there is no study on the quantitative changes of the proteomics and protein phosphorylation induced by exogenous nitrogen in plants under cadmium stress. Therefore, the main objective in the study was to investigate the effects of nitrogen on protein expression patterns in poplar plants under Cd stress. Thus, we performed comparative proteomic and protein phosphorylation analyses. The results further elucidate the important role of N in detoxifying plants with Cd in poplar species.

## 2. Results

### 2.1. Exogenous Nitrogen Reduces Cadmium Toxicity in Poplar Leaf Photosynthesis and Promotes Growth

No obvious morphological differences were seen at the beginning of the treatment period. However, as the treatment time was prolonged, the growth of Cd-treated plants was significantly inhibited, and their leaves’ colors changed from green to yellow green, and even leaf etiolation was obvious in Cd-treated plants by the end of the experiment. The level of chlorophyll is an important index that reflects the growth of a plant and the tolerance of that plant to Cd stress. Here, we identified that Cd treatment decreased the chlorophyll a (Chl a), chlorophyll b (Chl b) and total chlorophyll (Chl) content by 30.7%, 53.3% and 36.5%, respectively, when compared with the control plants (Table 1). Additionally, Cd stress obviously reduced the plants’ photosynthetic capabilities and Chl fluorescence. Cd treatment decreased the net photosynthetic rate (*Pn_max_*), transpiration rate (*R*), maximum quantum efficiency (*AQE*), stomatal conductance (*g_s_*) and maximum quantum efficiency of PS II (*F*v/*F*m) by 51.3%, 53.6%, 56.5%, 46.6% and 35.5%, respectively, when compared to the control plants (Table 2). The above data reveal that Cd stress severely inhibits growth.

Conversely, the N + Cd treated plants grew well; their leaves maintained their green color and even turned dark green as the treatments progressed. There was no significant difference in the Chl a, Chl b and total Chl between the N + Cd treated plants and the control plants (Table 1). On the other hand, Pn_max_, R, AQE, g_s_ and the ratio of Fv/Fm increased 55.6%, 51.9%, 66.7%, 58.1% and 33.5%, respectively, under N + Cd treatment compared to the sole Cd treatment. The above data reveal that Cd stress damaged the poplar plants’ viability. The addition of nitrogen to Cd treated plants may alleviate the suppression of Cd in poplar plants and promote growth.

### 2.2. Exogenous Nitrogen Can Enhance the Plants Absorption and Transportation of Cd

The Cd content in soil and in plants can effectively reflect the absorption ability of plant to Cd. As shown in Table 3, the Cd concentrations in soil, roots and leaves during Cd treatment or N + Cd treatment were observably higher than those of the control. Moreover, Cd content in roots was obviously higher than that in leaves, which indicates that Cd was mainly trapped in the root regions. In addition, Cd concentrations in the roots and in leaves under N + Cd treatment increased by 30.3% and 59.5%, respectively, while the Cd in soil decreased by 31.2% when compared to the Cd-stressed plants. The results reveal that the addition of N significantly enhanced the absorption and transportation of plants to Cd.

### 2.3. Exogenous Nitrogen Reduces Cadmium-Induced H_2_O_2_ and MDA Generation and Enhances Glutathione (GSH) and Phytochelatin (PCs) Accumulation

Cd exerts its toxic effects on plants mainly through the production of large amounts of reactive oxygen species (ROS) [35]. Exogenous nitrogen can mitigate the damaging effects of ROS [26]. We examined whether exogenous nitrogen could alleviate cadmium toxicity in poplar plants by maintaining the redox balance in cells. H_2_O_2_ reflects levels of cellular oxidation, and MDA is an end-product of lipid peroxidation. On the other hand, GSH and PCs reflects scavenging Cd^2+^ ability of plants. Therefore, we detected the H_2_O_2_, MDA, GSH and PCs levels in poplar leaves following Cadmium or Cd +N treatment (Table 4). The data show that H_2_O_2_, MDA, GSH and PCs significantly increased when plants were faced with Cd stress, while the addition of Cd +N decreased cadmium-induced H_2_O_2_ and MDA accumulation and obviously increased GSH and PCs accumulation.

### 2.4. Impacts of Cd or Cd + N on the Global Proteome of Poplar Plants

**Figure 1:**
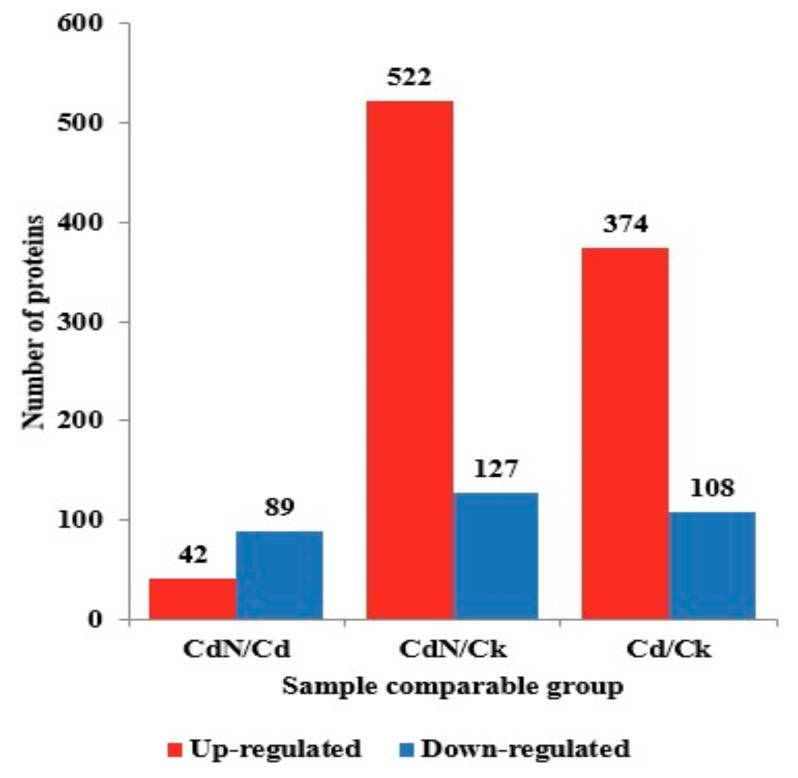
The differentially expressed proteins in the Cd/CK, Cd + N/Cd and Cd + N/CK groups.

In order to elucidate the important role of N in plants against Cd toxicity in poplar species, we measured the protein abundance for the following three groups: (1) Cd/CK, (2) Cd + N/Cd and (3) Cd + N/CK. The primary proteome data and phosphoproteome data were deposited in the PRIDE Archive (No. PXD013360). In this study, 6573 proteins were identified, and 5838 of them were quantified. With a fold-change threshold of > 1.3 and a *p*-value < 0.05, 375 and 108 proteins were up- and down-regulated, respectively, in the Cd/CK group; 42 and 89 proteins were up- and down-regulated, respectively, in the Cd + N/Cd group; and 522 and 127 proteins were up- and down-regulated, respectively, in the Cd + N/CK group (Figure 1).

**Figure 2:**
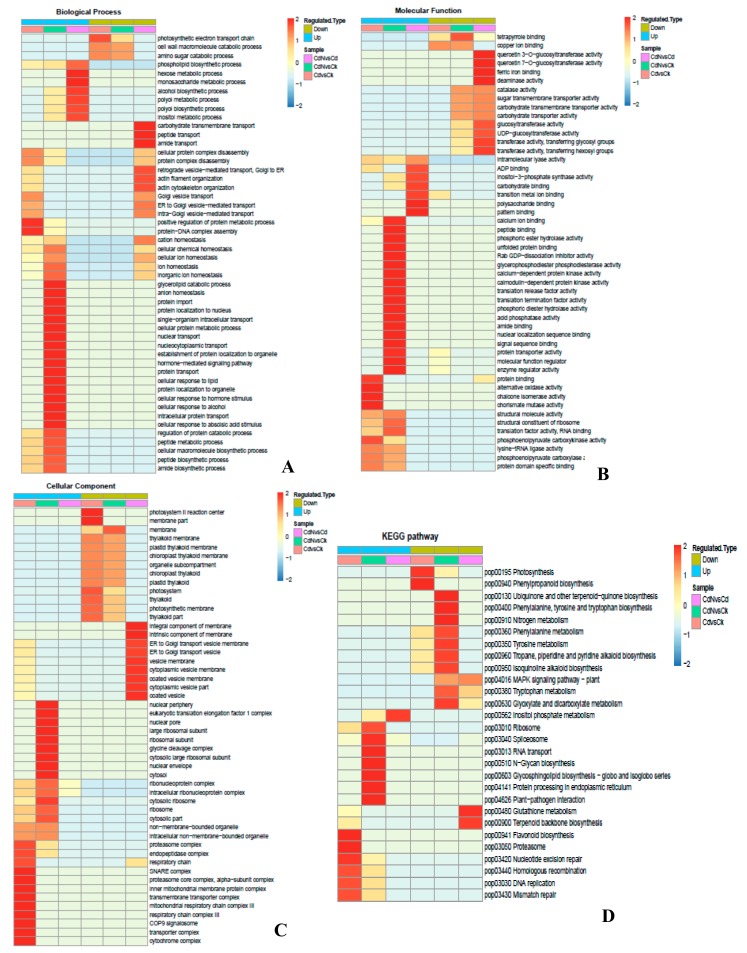
A comprehensive heatmap for cluster analysis of the enrichment patterns of differentially expressed proteins up-regulated and down-regulated based on their Z-score (–log10 (*p*-value); from –2 to 2). Cluster membership were visualized by a heat map using the heatmap.2” function from the “gplots” R-package. (**A**) A biological process category. (**B**) A molecular function category. (**C**) A cell component category. (**D**) A Kyoto Encyclopedia of Genes and Genomes (KEGG) pathway analysis for all differentially expressed proteins.

To characterize the functions of the differentially expressed proteins in the Cd/CK or Cd + N/Cd groups, gene ontology (GO) enrichment-based clustering analyses were performed (Figure 2). When comparing the differentially expressed proteins between the Cd treatment and CK, we found that, in the biological process category (Figure 2A), positive regulation of the protein metabolic process, protein-DNA complex assembly, cellular protein complex disassembly, protein complex disassembly and Golgi vesicle transport were highly enriched among the up-regulated proteins; down-expressed proteins were observed in the regulation of the photosynthetic electron transport chain, cell wall macromolecule and amino sugar catabolic processes. In the molecular function category (Figure 2B), the up-expressed proteins were mainly enriched in their protein binding, alternative oxidase activity, chalcone isomerase activity, chorismate mutase activity and phosphoenolpyruvate carboxykinase activity, while the down-expressed proteins mainly included copper ion binding and tetrapyrrole binding. In the cellular component category (Figure 2C), the up-expressed proteins were mainly enriched in 12 processes, including the cytochrome complex, transporter complex, COP9 signalosome, respiratory chain complex III, mitochondrial respiratory chain complex III, transmembrane transporter complex and others. Down-regulated proteins were primarily located in the photosystem II reaction center, membrane part, photosystem, thylakoid, photosynthetic membrane, thylakoid part, chloroplast thylakoid membrane and others.

However, when comparing the functions of the differentially expressed proteins between Cd + N and Cd treatment, we found that in the biological process category (Figure 2A), the inositol metabolic process, polyol biosynthetic process, polyol metabolic process, alcohol biosynthetic process, monosaccharide metabolic process, hexose metabolic process and phospholipid biosynthetic process were up-regulated in the Cd + N treatment compared to sole Cd stress, while carbohydrate transmembrane transport, peptide transport, amide transport, retrograde vesicle-mediated transport, actin filament organization, actin cytoskeleton organization and Golgi vesicle transport were down-regulated by exogenous nitrogen addition under Cd stress. In the molecular function category (Figure 2B), the up-expressed proteins were mainly involved in pattern binding, polysaccharide binding, transition metal ion binding, carbohydrate binding, inositol-3-phosphate synthase activity, ADP binding and intramolecular lyase activity, while the down-regulated proteins were mainly involved in quercetin glucosyltransferase activity, ferric iron binding, deaminase activity, transferase activity, transferase activity and glucosyltransferase activity. For the cellular component category, Figure 2C showed that the down-expressed proteins in plant cells with N + Cd treatment primarily consisted of the integral component of membrane, intrinsic components of the membrane, ER to Golgi transport vesicle membrane, vesicle membrane, cytoplasmic vesicle membrane, coated vesicle membrane, cytoplasmic vesicle part and coated vesicle.

The cellular pathways of the different proteins regulated by Cd stress or Cd + N treatments were profiled using gene ontology (GO) and the Kyoto Encyclopedia of Genes and Genomes (KEGG) database (Figure 2D). The flavonoid biosynthesis, proteasome, nucleotide excision repair, homologous recombination, DNA replication and mismatch repair pathways were up-regulated by Cd stress, while the photosynthesis and phenylpropanoid biosynthesis pathways were down-regulated by Cd stress. In addition, the inositol phosphate metabolism pathway was up-expressed in Cd + N treatment plants, while the glutathione metabolism and terpenoid backbone biosynthesis pathways were down-regulated by Cd + N treatment in comparison with sole Cd stress.

### 2.5. Impacts of Cd and Cd + N on the Protein Phosphorylation Levels in Poplar Plants

**Figure 3:**
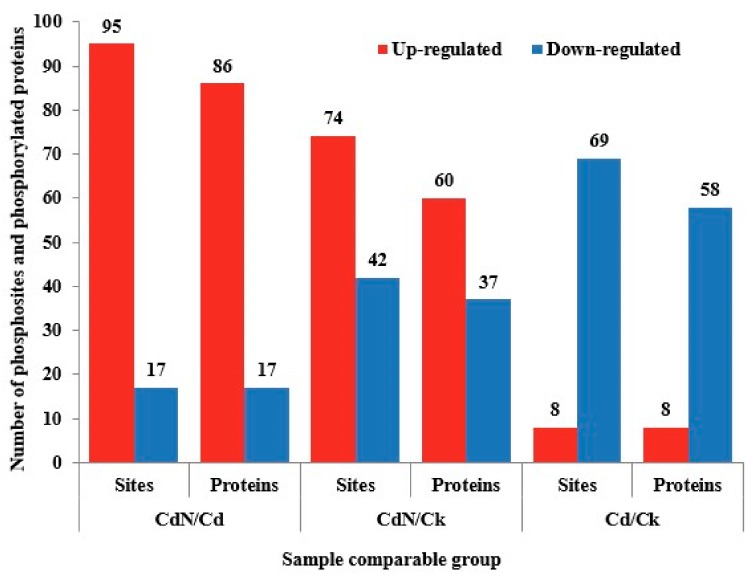
The differentially expressed phosphosites and phosphorylated proteins (after being normalized) in the Cd/CK, Cd + N/Cd and Cd + N/CK groups.

Protein phosphorylation is important in signal transduction systems, as it affects the activity of its target. In this study, the enrichment efficiency of phosphorylated peptides was 94.1% and the mean precursor ion fraction (PIF) value of phosphorylated peptides was 0.77. In addition, 7605 phosphosites in 3479 proteins were identified, and 5094 phosphosites in 2848 proteins were quantified based on a localization probability of > 0.75, according to the LC–MS/MS analysis. The phosphoproteome data were obtained after the proteomic difference was normalized. The results quantified 1471 phosphosites in 721 proteins. The differences in phosphosites and phosphorylated proteins upon Cd or Cd + N treatment were compared with the control and are shown in Figure 3. According to a fold-change threshold of > 1.2, and a *p*-value < 0.05, the Cd stress up-regulated eight phosphosites in eight proteins and down-regulated 69 phosphosites in 58 proteins in the Cd/CK group, whereas N + Cd treatment up-regulated 95 phosphosites in 86 proteins and down-regulated 17 phosphosites in 17 proteins when compared to sole Cd stress. In addition, N + Cd treatment up-regulated 74 phosphosites in 60 proteins and down-regulated 42 phosphosites in 37 proteins when compared to the control.

**Figure 4:**
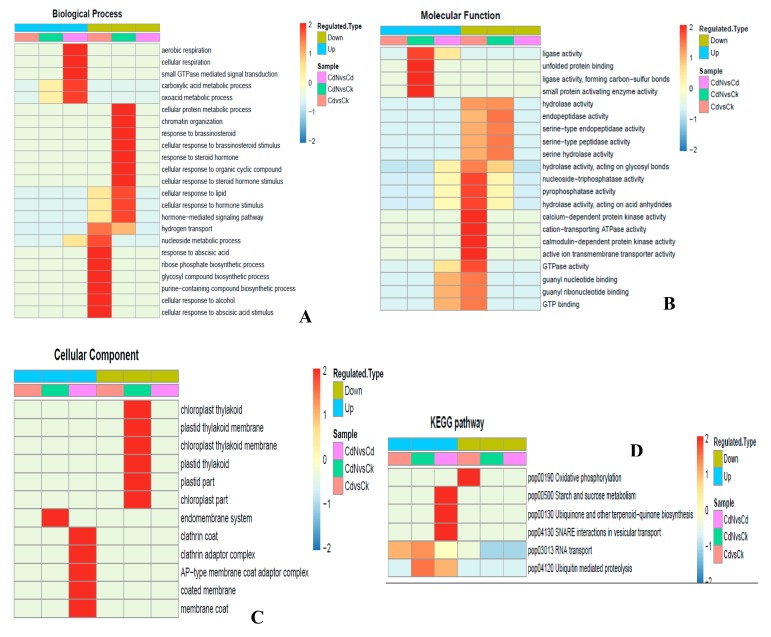
Gene ontology (GO) enrichment-based clustering analysis of the quantified phosphoproteome based on Zscore (–log10 (*p*-value); from –2 to 2). (**A**) biological process category. (**B**) The molecular function category. (**C**) The cell component category. (**D**) KEGG pathway analysis.

A GO enrichment method was performed to analyze the functions of the different phosphosites and the different phosphoproteins between Cd and Cd + N treatment. As shown in Figure 4A, in the biological process category, the process of the cellular response to abscisic acid stimulus, cellular response to alcohol, purine-containing compound biosynthesis, glycosyl compound biosynthesis, ribose phosphate biosynthesis, response to abscisic acid, nucleoside metabolic and hydrogen transport were down-regulated under Cd stress. For the molecular function cluster (Figure 4B), 17 processes including active ion transmembrane transporter activity, calmodulin protein kinase activity, cation transporting ATPase activity, calcium-dependent protein kinase activity, hydrolase activity, acting on acid anhydrides, pyrophosphatase activity and others, were down-regulated by Cd stress. However, there were no differentially expressed proteins enriched in the cellular component category (Figure 4C).

However, when characterizing the functions of the differentially phosphorylated proteins between Cd + N and Cd treatment, we found that aerobic respiration, cellular respiration, small GTPase mediated signal transduction, the carboxylic acid metabolic process and the oxoacid metabolic process were enriched in the biological process category and were up-regulated by exogenous nitrogen addition following Cd stress (Figure 4A). For the molecular function cluster (Figure 4B), GTP binding, guanyl ribonucleotide binding, guanyl nucleotide binding and GTPase activity were up-regulated in the Cd + N treatment compared to Cd stress alone. In addition, the differentially expressed proteins in response to Cd + N treatments were chiefly located in the membrane coat, coated membrane, clathrin adaptor complex, AP-type membrane coat adaptor complex and clathrin coat (Figure 4C).

The alterations in signaling pathways induced by the Cd or Cd + N treatments were analyzed using the KEGG clustering method (Figure 4D). The oxidative phosphorylation pathway was down-regulated by Cd stress. Interestingly, some signaling pathways, including starch and sucrose metabolism, ubiquinone and other terpenoid−quinone biosynthesis and SNARE interactions in vesicular transport were up-regulated by exogenous nitrogen addition following Cd stress, suggesting that the addition of exogenous nitrogen under Cd stress is beneficial to enhance the photosynthetic ability of plants and promote the poplar to absorb and transport cadmium through different pathways. The above results are consistent with those of previous analyses (Table 2 and Table 3).

## 3. Discussion

### 3.1. Cd Stress Induced Oxidative Injuries and Inhibited Photosynthesis

The photosynthetic capacity of plants is closely related to their growth. Cd stress seriously destroys the photosystem through the formation of ROS, which decreases the photosynthetic capacity and inhibits growth [27,28]. In the present study, Cd induced an accumulation of H_2_O_2_ and MDA (Table 4). Moreover, Cd ions inhibited the activity of RuBisCo and interfered with the reactions in photosystem I and photosystem II. For example, some proteins (A0A2K2BNL0 and B9MYU1) related to the photosystem I reaction pathway and one protein (A0A2K1Z195) associated with the photosystem II reaction pathway were down-regulated by Cd stress. In addition, one RuBisCo protein (A9PJ06), one ATP synthase CF0 A subunit protein (A0A2K1WNK6) and one thioredoxin protein (B9H8W5) showed a downward in expression with longer-term (60 d) of Cd stress (Table 5).

### 3.2. Plants Actively Exert a Range of Approaches in the Defense against Cadmium Stress

Plants actively exert a range of strategies to strengthen their tolerance to heavy metal exposure. First, they up-regulate the expression of stress-responsive proteins; second, they stimulate the activities of antioxidant enzymes to scavenge the cellular ROS induced by heavy metal stress; third, they up-regulate the expression of cell wall proteins to constraint more heavy metal ions and fourth, they increase the activities of enzymes associated with glutathione (GSH) and phytochelation (PC) to sequestrate more heavy metal ions.

In this study, stress-responsive proteins including two heat shock proteins (HSPs; HSP70, A0A2K2BZL0; HSP90, T2AUM9), three 14-3-3 proteins (A0A2K2BGF4, A9P8Q7 and A9PCV6), and five disease resistance proteins (Q6ZXH8, A0A2K1XHW1, A0A2K1YCK6, A0A2K2C6K6 and A0A2K1XHW1) were up-regulated in poplar plants when exposed to Cd treatment. Heat shock proteins are stress-responsive proteins and play an important role in protecting cells from elevated environments. CeHSP17 was significantly up-regulated by cadmium and zinc in wild-type *C. elegans* [36]. In transgenic *Arabidopsis*, PfHSP17.2 showed a higher resistant ability under heat, cold and salt stresses [37]. HSP20, HSP70 and HSP90 were increased in poplar leaves under Cd stress [38]. In the *Oxya chinensis*, OcHsp40, OcHsp70 and OcHsp90 were significantly up-regulated by acute Cd stress [39]. In this study, HSP70 and HSP 90 were up-regulated by Cd stress in *P*. *yunnanensis* leaves.

In higher plants, 14-3-3 proteins always bind to the signaling molecules and play crucial roles in stress responses and signal transduction [40]. In rice, the genes encoding the 14-3-3 protein was obviously up-regulated by arsenic stress [41]. In aluminum (Al)-tolerant soybean roots, the expression of 14-3-3 proteins were significantly enhanced by Al stress [42]. In the present study, three 14-3-3 proteins were up-regulated by Cd stress.

Interestingly, five bioresistance proteins were up-regulated by cadmium stress in the study, which reveal that the resistance protein may have dual functions including bio-resistance and abio-resistance.

The cell wall is confirmed to be the first barrier to protect plants against environmental stresses [43]. Many documents have reported that the cell wall can effectively bio-sorb Cd ions to alleviate their toxicity to plants [44]. Meyer et al. (2015) revealed that metal-tolerant plants could accumulate abundant Cd in cell walls [45]. The proteomics results in this study showed that one cinnamyl alcohol dehydrogenase (CAD) protein (A0A1L6K4D3) had higher expression levels after Cd stress. CAD catalyzes key steps in the pathway of lignin monomer biosynthesis. Up-expressed CAD can promote the synthesis of plant cell walls to resist various environmental stresses [46].

Antioxidant enzymes maintain cellular redox balance by scavenging reactive oxygen species (ROS) [47]. Cd stress mainly stimulates the production of ROS to damage plants. In this study, our proteomics results showed that one oxidoreductase protein (A0A2K1Z5Z6), one peroxisome protein (A0A2K1XV17) and one superoxide dismutase protein (B9ICD9) were significantly up-regulated by Cd stress. Thus, our results reveal that Cd stress actively regulates the expression of antioxidant proteins to balance the cellular redox level and minimize the damage of Cd to poplar plants.

### 3.3. Exogenous Nitrogen Alleviated the Toxicity of Cadmium to Poplar Plants

In the present study, the results reveal that Cd + N observably promotes poplar plant growth, improves chlorophyll content, reduces the accumulation of H_2_O_2_ and MDA, and enhances the absorption and transportation of plants to Cd. The differentially expressed proteins in Table 5 show that the positive interaction effects between Cd and N could partially explain how exogenous nitrogen effectively protects plants against Cd stress.

The growth of plants is positively related to photosynthesis. In this study, Cd stress significantly inhibited the photosynthesis of poplar plants. Eight proteins involved in photosynthesis showed lower expression levels when compared to the control. However, 15 proteins correlated to photosynthesis and energy metabolite were obviously up-regulated by Cd + N treatment, relative to the control values. Similarly, five photosynthetic proteins showed higher expression levels in N + Cd treated plants when compared to acute Cd stress. The differentially expressed ratio of Cd + N/CK for the proton gradient regulation (PGR5) protein was as high as 5.515. The data revealed that the expression of PGR5 was markedly up-regulated by the interaction of Cd + N. Previous studies have shown that PGR5 is closely related to the cyclic electron flow in PS I [48]. Paredes and Quiles [49] revealed that when Hibiscus plants are subjected to cold stress, the amount of PGR5 polypeptide increases, suggesting that under abiotic stress, up-expressed PGR5 could protect photosystems and enhance the resistance of plants to environmental stress. Therefore, the PGR5 protein (A0A2K2BVX5) was screened out as a candidate protein for further research.

Exogenous nitrogen addition could help poplar plants actively respond to cadmium stress, which was validated by comparing the differentially expressed proteins between Cd + N treatment and the control or between Cd + N treatment and acute Cd treatment. Our proteomics results show that 21 and 11 differentially stress-responsive proteins were observably up-regulated by N + Cd treatments when contrasted with CK, and sole Cd, respectively. These 21 proteins mainly included eleven heat shock proteins (HSPs), three 14-3-3 proteins, one TMV resistance protein, one mitogen activated protein kinase protein (MAPK), two proliferation-associated proteins 2G4 (PA2G4), one DnaJ protein and two translationally controlled tumor proteins (TCTP). Notably, the differentially expressed ratio of Cd + N/CK about MAPK, TCTP, 14-3-3, HSP70, DnaJ, and PA2G4 was as high as 2.968, 2.311, 3.609, 4.264, 5.113 and 6.879, respectively. The functions of HSP and the 14-3-3 in stress-response were explained in the previous section. Research has shown that MAPK plays an important role in cell differentiation and stress response. The activity of MAPK was induced by heavy metals in alfalfa and rice [50]. Existing studies have shown that plant TCTP is involved in environment stress signaling and that overexpression of *AtTCTP* reinforce drought tolerance, depending on the abscisic acid pathway [51]. Wang et al. [52] revealed that *OsTCTP* could significantly reduce the accumulation of the Hg-induced reactive oxygen species (ROS), thereby enhancing the rice plants’ tolerance to Hg stress. In this study, two TCTPs were up-regulated by N + Cd treatment. The DnaJ protein was reported to be important in maintaining cellular homeostasis under stress conditions to enhance plants’ resistance. For example, a DnaJ protein located in the chloroplast maintained its photosystem II stability and improved the resistance of plants to chilling stress [53]. However, the mechanism by which the DnaJ protein facilitates plant resistance to Cd stress remains unclear.

The existing studies reported that the PA2G4 protein actively regulates the DNA replication in eukaryotic cells. At present, studies on the functions of PA2G4 proteins have mainly focused on humans and animals, with few studies on plants. In our study, the differentially expressed ratio of Cd + N/CK of PA2G4 ranged to 6.879, thus indicating that exogenous nitrogen addition helps to maintain normal DNA replication in plant cells and alleviate cadmium toxicity in poplar plants.

Antioxidant enzymes may be crucial for alleviating oxidative stress [54]. Our proteomics results show that peroxiredoxin (Prx), peroxisome (POD) and glutathione transferase (GST) are observably up-regulated by N + Cd treatments when contrasted with CK or sole Cd treatment, respectively. The differential expression ratio of Cd + N/CK or Cd + N/Cd for Prx was up to 3.588 and 3.867, respectively. Peroxiredoxins (Prxs) are a family of ubiquitous proteins in all kingdoms of life. Prxs are important for antioxidant defense and participate in redox signaling. The inactivation of Prx by phosphorylation causes H_2_O_2_ accumulation [55]. In the present study, Cd + N treatment activated the expressions of Prx and obviously decreased the accumulation of H_2_O_2_, which agrees with the results of past studies.

When cadmium ions are absorbed by plant roots, some of the ions are transferred aboveground from the plant and are then chelated with glutathione (GSH) and phytochelation (PC) to be sequestrated in the vacuoles. In this study, the addition of N significantly enhanced the transportation and absorption of Cd in plants. The data for the proteomics also revealed that eleven binding proteins, seven transporter proteins and five storage proteins associated with Cd^2+^ were up-regulated by N + Cd treatments. Of the eleven binding proteins, four were calcium-binding proteins, three were GTP-binding proteins, two were DNA-binding proteins and two were metal ion-binding proteins. The transporter proteins up-regulated by N + Cd were mainly ABC transporter proteins, which accounted for 57.1% of the total differential transporters. ABC transporter proteins are widely found in living organisms and belong to the superfamily of transporters. Evidence in the existing literature has shown that ABC transporters are important for the detoxification of heavy metals [56]. In *Arabidopsis*, AtSTAR1 (an annotated ABC transporter) is involved in the basic detoxification of aluminum stress [57]. AtMRP3, an ABC transporter gene, shows a higher expression levels in Cd-treated plants [58]. In the present study, we found much higher levels of ABC transporter proteins induced by Cd + N, which suggests that exogenous nitrogen addition could enhance the activities of ABC transporter proteins to carry and transport more Cd ions. Among the differentially expressed storage proteins, four were vacuolar proteins and one was a bark storage protein, which reveals that exogenous nitrogen addition can increase the accumulation of cadmium in poplar plants.

Existing studies have shown that transcriptional regulation and translational regulation are the conserved strategies for plant responses to biological and abiotic stress [59]. The responses of plants to Cd stress are often modulated by transcription factors (TFs) and translation initiation factors (IFs). These transcription factors usually include basic helix-loop-helix (bHLH), WRKY, ERF, MYB, NAC, MADS, Zinc-Finger transcription factor (ZFPs), etc. For example, overexpression of *ThWRKY7* enhanced plant tolerance to cadmium stress [29]. ZAT6, belonging to the Zinc-Finger transcription factor family, positively regulates cadmium tolerance through the glutathione-dependent pathway in *Arabidopsis* [60]. In plants, the translation initiation factor (IFs) is the main eukaryotic translation initiation factor (eIF). Chou et al. [61] revealed that the expression of rice *eIF5A* genes, OseIF5A- and seIF5A-2 were remarkably up-regulated by salt and heavy metal stresses. In the present study, we found much higher levels of WRKY, ZFPs, TFs and eIFs in poplar plants under Cd + N treatment. These findings indicate that plant’s responses to Cd stress are positively regulated by exogenous nitrogen.

### 3.4. Exogenous Nitrogen Promoted Protein Phosphorylation to Enhance Poplar Plants’ Resistance to Cadmium Stress

Protein phosphorylation plays an important regulatory role in the immune system. Previous studies on the regulation of plant responses to stress by protein phosphorylation mainly focused on drought, cold, salinity and other biotic stress [62]. Moreover, the plant materials used were mainly model species, such as *Arabidopsis thaliana*, rice (*Oryza sativa*), etc. Heavy metal-responsive phosphoproteins may be involved in heavy metal (HM) uptake, HM transportation, HM sequestration and HM detoxification. However, there are few studies on the regulatory function of protein phosphorylation in woody plants responses to HM stress. Therefore, we are the first to investigate the protein phosphorylation changes in poplar plants under Cd stress or Cd + N treatment.

The differences in phosphorylated proteins under Cd or Cd + N treatment were compared with the control after the proteomic difference was normalized, and the results are shown in Figure 3. The changes in the trend for the phosphoproteome data were similar to those found in the proteomic data. Many proteins up-regulated by Cd + N treatment also had higher expression levels after phosphorylation (Table 6). In addition, both Cd stress and Cd + N treatment may affect oxidative phosphorylation. However, Cd + N treatment obviously lead to greater protein phosphorylation and more phosphorylated proteins being up-expressed, while sole Cd stress induced a greater number of phosphorylated proteins to be down-expressed. These proteins largely included zinc finger proteins (ZFPs), ABC transporter proteins (ABC), heat shock proteins (HSPs), translation initiation factors (IFs) and peroxidase (POD). Interestingly, eight sites in the splicing factor 3B subunit protein (SF3B1) (A0A2K2C6G4) were phosphorylated, and all of them were up-regulated under Cd + N treatment. In addition, three phosphosites in the HSP70 protein showed higher expression levels in Cd + N treated plants. SF3B1 is an mRNA splicing factor. Importantly, SF3B1 mutations have been detected in many cancers [63]. At present, studies on the important role of SF3B1 have mainly focused on humans and animals, while its role in plants has not been reported. Kim Guisbert et al. [64] revealed that SF3B1 regulates the activity of the heat shock transcription factor (HSF1) via mRNA splicing to enhance stress-sensitive abilities. In this study, eight phosphosites in the SF3B1 were up-expressed in Cd + N treated plants, which indicated that SF3B1 may modulate poplar plants’ resistant to Cd stress by mRNA splicing at the transcriptome. On the other hand, HSP70 is an important stress-responsive protein, and plays a vital role in stress responses against changes under extreme environments [65]. Assimon et al. [66] revealed that HSP70 autophosphorylation helps to bind its chaperone and further to realize its biological functions. Similarly, three phosphosites in HSP70 were all up-regulated by Cd + N treatment, which indicates the phosphorylated HSP70 may better exercise its function in protecting plant cells or tissues from Cd stress. However, in the study, the intriguing link between SF3B1 and Cd-response, as well as the variety of roles for both SF3B1 and HSP 70 in response to Cd stress, are still not clear, and deserve further study.

### 3.5. The Key Regulatory Proteins Play Their Important Roles in Plant Cellular Responses to Cd Stress

Plant cellular responses to heavy metal stress may involve complex interconnected networks of signaling pathways. Transcription factors (TFs) are proteins that have been identified their important roles in plant cellular responses to Cd stress. These TFs includes WRKY, MYB, NAC, HSF, ZIP, etc. [67]. In parallel with comparative proteomic and protein phosphorylation analyses, we performed transcriptome sequence using the same batch of materials. The transcriptomic data was not published, but can be checked in the National Center for Biotechnology Information (NCBI) Gene Expression Omnibus (GEO) Sequence Database (accession number GSE140398). The data of transcriptome reveal that exogenous nitrogen addition under Cd stress induces more differentially expressed TFs, such as WRKY, HSF, LOB, NAC, MYB, ZAT and bHLH. Among these TFs, three WRKYs, five ZATs and one HSF showed significantly higher expression levels in poplar plants under Cd + N treatment compared to sole Cd stress. At the same time, proteomic data show that one WRKY, three ZFPs belonged to ZAT family, and one HSP70 belonged to HSF family, were significantly up-regulated by exogenous nitrogen applied to Cd stress. Therefore, combine transcriptomic data with proteomic data, WRKY, HSP70 and ZFP proteins can be considered as the key candidate proteins for further study.

## 4. Conclusions

**Figure 5:**
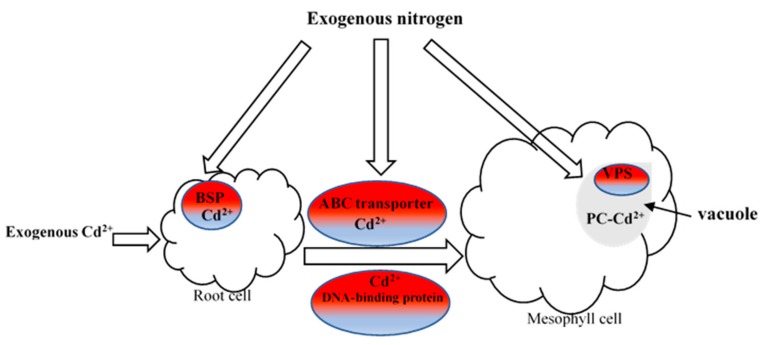
Exogenous nitrogen enhanced the uptake, transport and accumulation of cadmium in poplar plants by up-regulated the expressions of proteins. BSP, bark storage protein; VPS, vacuolar protein sorting-associated protein. Red, up-regulated protein.

**Figure 6:**
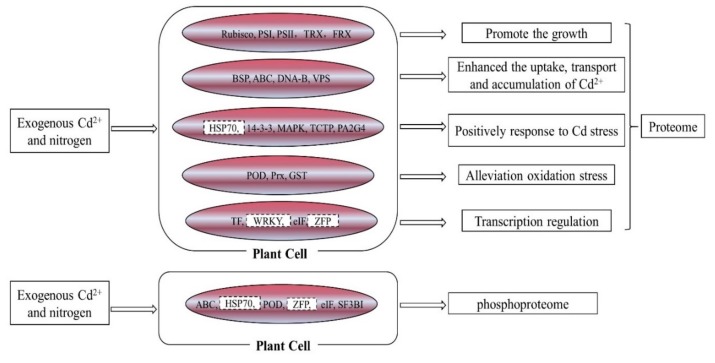
Exogenous nitrogen alleviated the toxicity of cadmium to poplar plants through multiple channels. Red, up-regulated protein; PS I, Photosystem I; PS II, photosystem II; TRX, thioredoxin family protein; FRX, ferredoxin family protein; ABC, ABC transporter protein; DNA-B, DNA-binding protein; HSP70, heat shock protein 70; 14-3-3, 14-3-3 protein; MAPK, Mitogen-activated protein kinase; TCTP, translationally controlled tumor-like family protein; PA2G4, proliferation-associated protein 2G4-like; POD, Peroxidase; Prx, Peroxiredoxin; GST, glutathione transferase; TF, transcription factor; elF, Eukaryotic translation initiation factor; ZFP, zinc finger protein; SF3BI, splicing factor 3B subunit 1-like. WRKY, ZFP and HSP70 were selected as the key regulation proteins.

Combing the proteomic data and phosphoproteomics data, we could deduce the reasons why exogenous nitrogen can enhance poplar plants’ resistance to cadmium and improve the phytoremediation efficiency of cadmium-contaminated soil. Exogenous nitrogen alleviated the toxicity of cadmium to poplar plants through multiple channels. First, the addition of nitrogen to Cd treated plants up-regulated the bark storage protein (BSP), ABS transporter protein, DNA-binding protein and vacuolar protein sorting-associated protein (VPS) to promote the uptake, transportation and accumulation of cadmium in poplar plants (Figure 5). Second, the addition of nitrogen induced the accumulation of glutathione (GSH) and phytochelatin and increased the activities of antioxidant enzymes to weaken the cadmium-induced damage to poplar plants (Table 4 and Table 5 and Figure 6). Third, the addition of nitrogen to Cd treated plants up-regulated the expressions of some proteins, such as the responsive proteins (HSP70, 14-3-3, MAPK, etc.), some transcription factors (WRKY, TF, ZFP, etc.), and eukaryotic translation initiation factor (elF), to strengthen the resistance of poplar plants to Cd stress. Lastly, phosphorylated SF3B1 and HSP70 possibly co-effected poplar plants’ resistance to cadmium stress. Taken together, the proteome and phosphoproteome data show that nitrogen serves a protective role in plants against Cd stress.

## 5. Materials and Methods

### 5.1. Plant Material and Treatment

The cuttings from *Populus yunnanensis* plants were used as the experimental materials and NH_4_HCO_3_ and CdCl_2_ as the major reagents in the study. Seventy-two healthy cuttings about 15 cm long were obtained from 15 Yunnan poplars (*Populus yunnanensis*). The *P*. *yunnanensis* samples were obtained from their native habitats in Butuo county of Liangshan prefecture, Sichuan province, China. Since the wide Yunnan poplar plants were not privately owned, and they were not listed among the endangered species, specific permits were not required for this study. Moreover, the selected trees shared the same habitat. After sampling, these 72 cuttings were replanted into 24 plastic pots (three cuttings per pot), and each pot was filled with 15 kg of dry soil. The diameter and deep of the pot were 37 and 28 cm, respectively. The basic characteristics of the cultivation soil were as following: pH 5.8, total organic matter 14.23 g·kg^−1^, total nitrogen 0.58 g·kg^−1^, total phosphorus 0.42 g·kg^−1^, total potassium 2.35 g·kg^−1^ and total cadmium 0.785 mg·kg^−1^. The pots with Yunnan poplar cuttings were placed in a chamber with a 16 h photoperiod, a 25/20 °C temperature, and 70% air humidity. A polyethylene disc under each pot was used to avoid run-off. When the plants were 20 cm in height with an average of 10–15 leaves, the 24 pots were divided into three sets. One set acted as a control, and the second set was treated with Cd. Meanwhile, the third set was treated with Cd and N. The method of cadmium treatment was based on the phenomenon of the gradual infiltration of cadmium into soil in nature. A total of 1.22 g CdCl_2_·2.5H_2_O and 2.52 g NH_4_HCO_3_ (equal to 40 mg Cd^2+^ ·kg^−1^ and 30 mg N·kg^−1^ dry weight soil) was added to each pot every 5 days. The amount of Cd and N added each time was based on the results from the preliminary test and our published research [30]. After 60 d of treatment, the fourth, fifth and sixth leaves from the apex of each seedling were sampled and immediately kept frozen at −80 °C for the subsequent analysis.

### 5.2. Chlorophyll Content and Cd Assays

Chlorophyll content was quantified according to the method of Lichtenthaler [68]. The Cd concentrations in soil, root and leaf tissue were detected using an inductively coupled plasma-optical emission spectrometer (ICP-OES; OPTIMA 2000A, PerkinElmer Co., Massachusetts, USA) after wet-digesting in HNO_3_–H_2_SO_4_–HClO_4_ [69]. Three independent biological replicates for each treatment and three technical repeats for each biological replicate were arranged to ensure the reproducibility.

### 5.3. Gas-Exchange Parameters and Chl Fluorescence Measurements

The fully expanded fourth, fifth and sixth leaves (from the apex) of the treatment and the control were used to monitor the photosynthetic rate and Chl fluorescence parameters. Gas-exchange parameters were quantified using a portable photosynthesis system (LI-6400, LI-COR Co., Nebraska, USA) according to the methods described by Zhang et al. [30]. Chlorophyll fluorescence parameters, such as the minimal fluorescence yield (*F*_0_), the maximal fluorescence yield (*F*_m_), the minimal fluorescence (*F*_0_’) and maximal fluorescence (*F*_m_’), were obtained according to the methods previously described [11]. The gas-exchange and fluorescence measurements were conducted on the same parts of the leaves.

### 5.4. H_2_O_2_, MDA (Malondialdehyde), Total GSH (Glutathione) and PCs (Phytochelatin) Content Analysis

H_2_O_2_ levels and MDA were measured as described previously [70]. The contents of GSH and PCs were quantified using the method of Gupta et al. [71]. GSH content was evaluated based on its absorbance at 412 nm. PC production was evaluated according to the D-value between non-protein thiols (NPT) and GSH [72]. The NPT was estimated as described by Ellman [73]. Three biological repeats were used to assess the content of these indexes.

### 5.5. The Differentially Expressed Proteins and Phosphorylated Proteins Analysis

The tandem mass tag (TMT) technique was applied to quantify the differentially expressed proteins in poplar leaves. TMT and IMAC (immobilized metal affinity chromatography) were used to identify the differentially phosphorylated proteins. Three independent biological replicates for each treatment and three technical repeats for each biological replicate were performed for assessment the proteome and protein phosphorylation levels.

#### 5.5.1. Protein Extraction, Trypsin Digestion, TMT Labeling and HPLC Fractionation

Protein extraction, trypsin digestion, TMT labeling and HPLC fractionation were performed using the procedure described by Yang et al. [74], with minor modifications. Leaf samples were grinded by liquid nitrogen into powder and then transferred to a centrifuge tube (5 mL). After that, four volumes of lysis buffer (8 M urea, 1% Triton-100, 10 mM dithiothreitol and 1% protease inhibitor cocktail) were added to the cell powder, followed by sonication three times on ice using a high intensity ultrasonic processor (Scientz). The remaining debris was removed by centrifugation at 20,000× *g* at 4 °C for 10 min. Finally, the protein was precipitated with cold 20% TCA for 2 h at –20 °C. After centrifugation at 12,000× *g* 4 °C for 10 min, the supernatant was discarded. The remaining precipitate was washed with cold acetone three times. The protein was then redissolved in 8 M urea and the protein concentration was determined with a BCA kit, according to the manufacturer’s instructions.

For digestion, the protein solution was reduced with 5 mM dithiothreitol for 30 min at 56 °C and alkylated with 11 mM iodoacetamide for 15 min at room temperature in darkness. The protein sample was then diluted by adding 100 mM TEAB to a urea concentration of less than 2 M. Finally, trypsin was added at a 1:50 trypsin-to-protein mass ratio for the first digestion overnight at 37 °C, and a 1:100 trypsin-to-protein mass ratio was used for a second 4 h-digestion to complete the digestion cycle.

After trypsin digestion, the peptide was desalted by a Strata XC 18 SPE column (Phenomenex) and vacuum-dried. The peptide was reconstituted in 0.5 M TEAB and processed according to the manufacture’s protocol for the TMT kit/iTRAO kit. Briefly, one unit of TMT kit/iTRAO reagent was thawed and reconstituted in in acetonitrile. The peptide mixtures were then incubated for 2 h at room temperature and pooled, desalted, and dried by vacuum centrifugation.

The dried peptides were fractionated by high pH reverse-phase HPLC using a Thermo Betasil C18 column (5 μm particles, 10 mm ID, 250 mm length) (Vigorous Co., USA). Briefly, peptides were first separated with a gradient of 8%–32% acetonitrile (pH 9.0) over 60 min into 60 fractions. Then, the peptides were combined into 18 fractions and dried by vacuum centrifugation.

#### 5.5.2. IMAC Enrichment

The phosphopeptide was enriched by immobilized metal affinity chromatography (IMAC) as previously described by Chen et al. [60]. Peptide mixtures were first incubated with an IMAC microsphere suspension with vibrations in a loading buffer, including 50% acetonitrile (CAN) and 6% trifluoroacetic acid (TFA). The IMAC microspheres with enriched phosphopeptides were collected by centrifugation (4 °C, 5000× *g* for 10 min), and the supernatant was removed. To remove nonspecific adsorbed peptides, the IMAC microspheres were washed with 50% acetonitrile/6% trifluoroacetic acid and 30% acetonitrile/0.1% trifluoroacetic acid, sequentially. To elute the enriched phosphopeptides from the IMAC microspheres, an elution buffer containing 10% NH_4_OH was added and the enriched phosphopeptides were eluted with vibration. The supernatant containing phosphopeptides was collected and lyophilized for LC–MS/MS analysis.

#### 5.5.3. LC–MS/MS Analysis

The tryptic peptides were analyzed using LC–MS/MS, as described by Yang et al. [74]. The enriched phosphopeptides were analyzed on a Q Exactive^TM^ Plus hybrid quadrupole-Orbitrap mass spectrometer (Thermo Fisher Scientific, Waltham, MA, USA), as described by Chen et al. [60]. The tryptic peptides were dissolved in 0.1% formic acid (solvent A) and directly loaded onto a home-made reversed-phase analytical column (15-cm length, 75 μm i.d.). The gradient comprised an increase from 6% to 23% for solvent B (0.1% formic acid in 98% acetonitrile) over 26 min, 23%–35% in 8 min and climbing to 80% in 3 min before holding at 80% for the last 3 min, all at a constant flow rate of 400 nL/min on an EASY-nLC 1000 UPLC system.

The peptides were subjected to NSI source analysis followed by tandem mass spectrometry (MS/MS) in a Q ExactiveTM Plus (Thermo Fisher Scientific, Waltham, MA, USA) coupled online to the UPLC. The electrospray voltage applied was 2.0 kV. The m/z scan range was 350–1800 for the full scan, and intact peptides were detected in the Orbitrap at a resolution of 70,000. Peptides were then selected for MS/MS using an NCE setting of 28, and the fragments were detected in the Orbitrap at a resolution of 17,500. A data-dependent procedure that alternated between one MS scan followed by 20 MS/MS scans with 15.0 s dynamic exclusion. The automatic gain control (AGC) was set at 5E4. The fixed first mass was set as 100 m/z.

#### 5.5.4. MS/MS Data Search

The resulting MS/MS data were processed using MaxQuant (v.1.5.2.8) (Max Planck Institute of Biochemistry). Tandem mass spectra were searched against the *Populus trichocarpa* protein database concatenated with a reverse decoy database. Trypsin/P was specified as cleavage enzyme allowing up to four missing cleavages. The mass tolerance for precursor ions was set as 20 ppm in first search and 5 ppm in the main search, and the mass tolerance for fragment ions was set as 0.02 Da. Carbamidomethyl on Cys was specified as a fixed modification, and phosphorylation modification and oxidation on Met were specified as variable modifications. False discovery rate (FDR) thresholds were adjusted to < 1%, and the minimum score for modified peptides was set as > 40.

#### 5.5.5. Relatively Quantified Phosphopeptides

The phosphopeptides were relatively quantified after the database search. For TMT quantification, the ratios of the TMT reporter ion intensities in MS/MS spectra (m/z 126–131) from raw data sets were used to calculate fold changes between samples. Only peptides unique for a given protein were considered for relative quantitation. For each sample, the quantification was normalized using the average ratio of all the unique peptide. Protein quantitation calculated from the median ratio of protein corresponding unique peptides when there were at least two unique peptides in a protein. To evaluate changes in differential abundance of protein, one-sample two-sided *t*-tests were performed with unique peptide ratio of protein corresponding. In general, a significance level of 0.05 was used for statistical testing, and we reported the *p* value or significance level any time a statistical test was performed. For overall group comparison, we regarded three replicate samples as one sample. In brief, the average reporter ion intensities of three replicate samples represented overall sample intensities. Then, we recalculated fold changes and statistics tests between samples.

#### 5.5.6. Bioinformatics Analysis

Gene ontology (GO) annotation was applied to determine the protein categories that were enriched. If some identified proteins were not annotated by the UniProt-GOA database, then InterProScan soft (The Minnesota Supercomputing Institute) was used to annotate the protein’s GO function based on the protein sequence alignment method. Then, the proteins were classified by gene ontology annotation based on three categories: biological process, cellular component and molecular function. Identified proteins domain functional description were annotated by InterProScan (a sequence analysis application) based on the protein sequence alignment method, and the InterPro domain database (http://www.ebi.ac.uk/interpro/) was used.

The pathways of differentially-expressed proteins and phosphoproteins were determined using KEGG tools-first, by using the KEGG online service tool KAAS to annotated the protein’s KEGG database descriptions and then by mapping the annotation results on the KEGG pathway database using the KEGG online service tools KEGG mapper.

#### 5.5.7. The Heat Map of Different Protein Functional Classification

The heat map of different protein functional classification was figured. We first collated all the categories obtained after enrichment along with their *p* values, and then filtered for those categories, which were at least enriched in one of the clusters with *p* value < 0.05. This filtered *p* value matrix was transformed by the function x = −log10 (*p* value). Finally, these x values were z-transformed for each functional category. These z scores were then clustered by one-way hierarchical clustering in Genesis. Cluster membership was visualized by a heat map using the “heatmap.2” function from the “gplots” R-package.

### 5.6. Statistical Analysis

Data were analyzed using SPSS statistical software, version 20.0 (IBM Co., Armonk, New York, USA). Results were evaluated by one-way ANOVA using a general model procedure followed by Tukey’s HSD post-hoc test. The results were presented as means ± SD, and a probability at *p* ≤ 0.05 was considered significant.

## Figures and Tables

**Table 1:** Interactive effects of Cd and N treatment on the chlorophyll content in poplar leaves (unit: mg/g FW).

Treatments	Chl a	Chl b	Total Chl
CK	1.653 ± 0.021 A	0.571 ± 0.013 A	2.224 ± 0.017 A
Cd	1.146 ± 0.014 C	0.267 ± 0.007 B	1.413 ± 0.011 B
N + Cd	1.521 ± 0.039 B	0.629 ± 0.026 A	2.150 ± 0.033 A

CK is the control, Cd indicates cadmium added and N + Cd indicates both nitrogen and cadmium added. Each value is the mean ± SD of three replicates, and the terms A, B, C and D within each column indicate significant differences between the means (*p* ≤ 0.05). The same is true below.

**Table 2:** Effects of cadmium by supplementing nitrogen on photosynthesis and Chl fluorescence in poplar plants.

Treatments	*Pn_max_* (μmolCO_2_·m^−2^s^−1^)	*R* (μmolCO_2_·m^−2^s^−1^)	*AQE* (molCO_2_·m^−1^ photon)	*g_s_* (mmol·m^−2^s^−1^)	*F*v/*Fm*
CK	14.73 ± 0.25 A	5.21 ± 0.19 A	0.62 ± 0.002 A	0.58 ± 0.001 A	0.823 ± 0.012 A
Cd	7.17 ± 0.34 C	3.39 ± 0.23 B	0.27 ± 0.004 C	0.31 ± 0.002 B	0.531 ± 0.007 C
N + Cd	11.16 ± 0.51 B	5.15 ± 0.32 A	0.45 ± 0.002 B	0.49 ± 0.003 A	0.709 ± 0.004 B

Pnmax, the net rate of photosynthesis; R, transpiration rate; AQE, maximum quantum efficiency; gs, stomatal conductance; Fv and Fm, maximum variable fluorescence in dark- and light-adapted leaves, respectively; Fv/Fm, maximum quantum efficiency of PS II.

**Table 3:** The application of nitrogen under Cd stress may significantly increase cadmium absorption by poplar plants from soil (unit: mg·kg^−1^).

Treatments	Cd in Soil	Cd in Root	Cd in Leaves
CK	0.68 ± 0.002 C	2.77 ± 0.05 C	1.41 ± 0.02 C
Cd	221.19 ± 3.42 A	128.56 ± 5.21 B	78.09 ± 2.58 B
N + Cd	152.13 ± 2.45 B	167.50 ± 5.65 A	124.52 ± 3.52 A

**Table 4:** Effects of cadmium supplementation with nitrogen on H_2_O_2_, MDA, GSH and PCs.

Treatments	H_2_O_2_ (nmol·g^−1^·FW)	MDA (μmol·g^−1^·FW)	GSH (nmol·g^−1^·FW)	PCs (nmol·g^−1^·FW)
CK	52.9 ± 3.4 C	9.24 ± 1.2 C	24.6 ± 1.27 C	121.3 ± 5.45 C
Cd	452.7 ± 5.9 A	22.99 ± 1.4 A	52.7 ± 2.41 B	325.9 ± 6.42 B
N + Cd	132.1 ± 3.6 B	13.21 ± 1.5 B	106.4 ± 3.45 A	543.4 ± 4.76 A

**Table 5:** List of partial proteins differentially regulated by Cd and Cd + N in poplar plants determined by LC–MS/MS analysis.

**Accession Number**	**Proteins Name**	**Cd/CK Ration**	**Regulated Type**	**Cd/Ck *p* Value**
Photosynthesis and energy metabolite				
A0A2K1Z195	photosystem II CP43 reaction center protein-like	0.639	down	0.014
A0A2K1WNK6	ATP synthase CF0 A subunit (chloroplast)	0.71	down	0.026
A9PJ06	ribulose bisphosphate carboxylase/oxygenase activase, chloroplastic isoform X1	0.747	down	0.037
B9H8W5	thioredoxin-like 2, chloroplastic	0.634	down	0.015
A0A2K2BNL0	“Photosystem I reaction center subunit XI family protein	0.631	down	0.033
B9MYU1	photosystem I subunit O-like	0.627	down	0.034
A0A2K1X1I0	outer envelope pore protein 37, chloroplastic-like	0.723	down	0.007
A9PF53	chaperone protein ClpB3, chloroplastic-like	0.739	down	0.0173
Response to stress				
A0A2K2BZL0	heat shock protein 70	2.171	up	0.023
A0A2K2BGF4	14-3-3 protein	1.372	up	0.032
A9P8Q7	14-3-3-like family protein	2.068	up	0.028
A9PCV6	14-3-3-like family protein	1.806	up	0.018
T2AUM9	HSP90	1.331	up	0.0201
Q6ZXH8	Putative pathogenesis-related protein	2.287	up	0.0225
A0A2K1XHW1	TMV resistance protein N	1.324	up	0.016
A0A2K1YCK6	putative disease resistance protein RGA4 isoform X4	1.71	up	0.029
A0A2K2C6K6	NBS-like putative resistance family protein	1.415	up	0.021
A0A2K1XHW1	TMV resistance protein	1.324	up	0.016
A0A1L6K4D3	Cinnamyl alcohol dehydrogenase (CAD)	3.77	up	0.010
DNA and ion binding				
A0A2K1WMZ6	DNA-binding family protein	2.176	up	0.025
A0A2K1XEE1	nucleotide-binding protein	1.629	up	0.001
A0A2K1XN19	oxidoreductase/transition metal ion-binding protein	1.393	up	0.048
A0A2K1Y9H8	DNA-binding protein	1.619	up	0.026
A9P929	DNA-binding family protein	1.365	up	0.014
A9PCK0	DNA-binding family protein	1.842	up	0.021
B9I6G6	calcium-binding EF hand family protein	1.487	up	0.003
U5GT53	DNA-binding family protein	1.34	up	0.011
A0A2K1XU09	zinc finger family protein	1.577	up	0.004
A9PEW2	zinc finger CCCH domain-containing protein	1.342	up	0.014
Transporters related to cadmium transport				
A0A2K1Z3W9	probable cadmium/zinc-transporting ATPase HMA1	1.321	up	0.014
A0A2K2ADM8	ABC transporter family protein	1.513	up	0.016
A9P875	copper transport protein CCH	1.352	up	0.001
A9P8F9	Copper-transporting ATPase RAN1 family protein	1.342	up	0.049
B9GJX7	ABC transporter family protein	1.442	up	0.013
B9GTB1	sugar transporter family protein	1.575	up	0.031
B9HIU2	sugar transporter family protein	1.751	up	0.028
Antioxidant activity				
A0A2K1Z5Z6	oxidoreductase family protein	1.324	up	0.014
A0A2K1XV17	peroxisome biogenesis protein 6	1.381	up	0.0461
B9ICD9	“superoxide dismutase [Fe], chloroplastic isoform X2	1.338	up	0.0282
**Accession Number**	**Proteins Name**	**Cd + N/Cd Ration**	**Regulated Type**	**Cd + N/Cd *p* Value**
Photosynthesis and energy metabolite				
A0A2K2BLH9	probable glutamyl endopeptidase, chloroplastic	1.336	up	0.039
U7E2H1	probable starch synthase 4, chloroplastic/amyloplastic isoform X2	1.634	up	0.004
B9HQD5	rubisco subunit binding-protein alpha subunit	1.359	up	0.029
Q3LUR8	Glyceraldehyde-3-phosphate dehydrogenase	1.484	up	0.002
B9GHJ1	thioredoxin family protein	1.859	up	0.013
Response to stress				
A0A2K1XHW1	TMV resistance protein N	1.353	up	0.015
A0A2K1Y4T9	signal recognition particle 14 kDa family protein	1.307	up	0.008
A0A2K1YPP0	disulfide isomerase family protein	2.338	up	0.002
A0A2K2BTY0	vacuolar-sorting receptor 6-like	1.837	up	0.012
A0A2K2CBE6	probable disease resistance protein At4g27220	1.342	up	0.032
B9GVR1	stress inducible family protein	1.431	up	0.015
B9HKA3	6a-hydroxymaackiain methyltransferase family protein	1.301	up	0.021
B9HSN8	UDP-N-acetylglucosamine pyrophosphorylase family protein	1.954	up	0.048
A0A2K2AYX4	ATP-dependent RNA helicase family protein	1.509	up	0.043
A0A2K2C8D8	DEAD-box ATP-dependent RNA helicase 46	1.405	up	0.047
B9HX26	huntingtin-interacting protein K-like	2.393	up	0.013
Transporters related to Cadmium transport				
A0A2K1ZUT7	oligopeptide transporter family protein	1.358	up	0.003
B9HXP4	vesical transport v-SNARE 12 family protein	2.084	up	0.025
A9PJD4	transmembrane protein	2.243	up	0.032
Antioxidant activity				
K9MCB1	Catalase	1.647	up	0.018
A0A2K1ZES8	Peroxiredoxin family protein (Prx)	3.867	up	0.004
A0A193KWX3	Glutathione S-transferase	1.731	up	0.036
A0A193KWY1	Glutathione S-transferase	1.642	up	0.014
Q5CCP3	Glutathione S-transferase	1.481	up	0.006
Regulation				
A0A2K2C504	transcription initiation factor TFIID subunit 15b-like	1.359	up	0.024
A0A2K2B8L4	zinc finger protein At1g67325-like isoform X1 (ZFPs)	1.634	up	0.032
A0A2K1YFZ8	dof zinc finger protein DOF1.4-like (ZFPs)	1.784	up	0.002
**Accession Number**	**Proteins Name**	**Cd + N/CK Ration**	**Regulated Type**	**Cd + N/CK *p* Value**
Photosynthesis and energy metabolite				
Q3LUR8	Glyceraldehyde-3-phosphate dehydrogenase	1.42	up	0.002
A9PFQ2	ribulose bisphosphate carboxylase/oxygenase activase, chloroplastic-like isoform X1	1.476	up	0.014
A9PJF4	Ribulose bisphosphate carboxylase/oxygenase activase family protein	1.371	up	0.032
B9HQD5	rubisco subunit binding-protein alpha subunit	1.392	up	0.029
B9I5M2	rubisco accumulation factor 1, chloroplastic	2.213	up	0.007
A0A2K2C7R0	Photosystem I reaction center subunit XI family protein	2.657	up	0.001
A9PEL0	photosystem II 11 kDa family protein	1.526	up	0.014
A9PFW0	photosynthetic NDH subunit of subcomplex B 4	2.815	up	0.004
U5GXD4	phosphoenolpyruvate carboxylase family protein	1.351	up	0.012
A0A0U1XA51	Phosphoenolpyruvate carboxylase	1.648	up	0.002
B9GHJ1	thioredoxin family protein (TRX)	2.181	up	0.023
A0A2K2B424	ferredoxin family protein (FRX)	1.657	up	0.031
A0A2K2B297	cytochrome c oxidase family protein	2.27	up	0.004
A0A2K2BVX5	PGR5-like protein 1A, chloroplastic	5.151	up	0.006
A0A2K2B5R5	PGR5-like protein 1A	2.849	up	0.014
Response to stress				
A0A2K1WUP1	HSP-interacting protein	1.92	up	0.024
B9HBT8	hsp70 nucleotide exchange factor fes1-like	4.264	up	0.017
A0A2K1YTL5	heat shock family protein	2.386	up	0.019
A0A2K2BZL0	heat shock protein 70	2.46	up	0.038
B9HMG7	heat shock protein 70 cognate	2.509	up	0.010
B9HMG8	heat shock protein 70 cognate	2.533	up	0.012
B9HTJ7	heat shock protein 70	1.913	up	0.013
B9HV59	heat shock protein 70	1.421	up	0.046
B9N9W5	heat shock protein 70 cognate	1.972	up	0.012
B9NBF4	heat shock protein 70 cognate	2.374	up	0.007
U5G4Y8	heat shock cognate 70 kDa protein 2-like	3.306	up	0.015
B9HKN2	DnaJ family protein	5.113	up	0.005
A0A2K2BGB8	14-3-3-like protein GF14 omicron	3.609	up	0.021
A9PBC6	14-3-3-like protein GF14 omicron	3.217	up	0.048
A9PCV6	14-3-3-like family protein	1.579	up	0.035
A0A2K1XHW1	TMV resistance protein N	1.791	up	0.003
A0A2K2BWJ4	Mitogen-activated protein kinase (MAPK)	2.968	up	0.012
A9PK38	translationally controlled tumor-like family protein (TCTP)	2.311	up	0.015
B9NAI3	translationally controlled tumor-like family protein (TCTP)	2.239	up	0.014
B9IGC6	proliferation-associated protein 2G4-like (PA2G4)	6.879	up	0.011
A0A2K2C4E6	proliferation-associated protein 2G4-like (PA2G4)	2.238	up	0.035
Transporters related to cadmium transport				
A0A2K1X4J9	ABC transporter G family member 22 isoform X2	1.582	up	0.0211
A0A2K2ADM8	ABC transporter family protein	1.658	up	0.010
B9HGA2	ABC transporter family protein	1.481	up	0.002
B9HQM5	ABC transporter family protein	1.511	up	0.042
A0A2K2C2F2	calcium-transporting ATPase 4, endoplasmic reticulum-type-like	2.026	up	0.007
A9P875	copper transport protein CCH	1.55	up	0.013
A9P8F9	Copper-transporting ATPase RAN1 family protein	1.427	up	0.041
Antioxidant activity				
A0A2K1XV17	peroxisome biogenesis protein 6 (POD)	1.534	up	0.004
A0A2K1YAM2	peroxisomal membrane protein PEX14-like isoform X2	1.94	up	0.011
A0A2K1ZES8	Peroxiredoxin family protein ((Prx)	3.588	up	0.005
B9NBW2	glutathione transferase (GST)	1.447	up	0.029
D2WL67	glutathione transferase GST	1.373	up	0.002
DNA and ion binding				
A0A2K1XEJ1	oxidoreductase/transition metal ion-binding protein	1.726	up	0.038
A0A2K1XN19	oxidoreductase/transition metal ion-binding protein	1.449	up	0.037
A0A2K1XB60	GTP-binding protein TypA/BipA homolog	1.388	up	0.025
A0A2K2CA16	calcium-binding EF-hand protein	1.734	up	0.016
A9P926	GTP-binding protein beta chain	1.533	up	0.013
A9P929	DNA-binding family protein	1.446	up	0.002
A9PCK0	DNA-binding family protein	1.626	up	0.008
A9PCU6	calcium-binding EF hand family protein	2.142	up	0.007
B9I2F7	calcium binding family protein	3.052	up	0.021
B9I2G9	GTP-binding protein beta chain	1.448	up	0.026
B9I6G6	calcium-binding EF hand family protein	1.43	up	0.029
Storage protein				
A0A2K1Y5T5	vacuolar protein sorting-associated protein 18 homolog (VPS)	1.581	up	0.008
A0A2K2BLH8	vacuolar protein sorting-associated protein (VPS)	1.625	up	0.039
A0A2K2BTY0	vacuolar-sorting receptor 6-like (VPS)	2.753	up	0.0001
U5GBE7	Vacuolar protein sorting-associated protein 35 (VPS)	2.391	up	0.019
A9PGW6	bark storage protein B-like (BSP)	4.043	up	0.018
Regulation				
A0A2K1XQU0	transcription factor 4G-like (TF4G)	2.062	up	0.003
A0A2K1XWY2	WRKY transcription factor 1 (WRKY1)	1.34	up	0.001
A0A2K1YGZ3	eukaryotic translation initiation factor 4E family protein (elF4E)	2.135	up	0.032
A0A2K1Z7J5	Translation initiation factor IF-3 (eIF3)	1.751	up	0.042
A0A2K2BI09	transcription factor (TF)	3.313	up	0.019
A0A2K2CCU4	translation initiation factor IF-2 family protein (eIF2A)	3.319	up	0.023
A9PC51	eukaryotic translation initiation factor 4G isoform X1 (elF4G)	1.64	up	0.029
B9GP88	eukaryotic translation initiation factor 4G isoform X1 (elF4G)	1.518	up	0.005
M9Z3T5	Eukaryotic translation initiation factor 5A (eIF5A)	1.816	up	0.002
M9ZCJ4	Eukaryotic translation initiation factor 5A (eIF5A)	2.299	up	0.006
A0A2K1XU09	zinc finger family protein (ZFPs)	1.671	up	0.007
A9PCM2	zinc finger protein GIS2-like (ZFPs)	1.686	up	0.004
U5FQR8	zinc finger matrin-type protein 2 (ZFPs)	1.485	up	0.026

**Table 6:** List of partial phosphorylated proteins differentially regulated by Cd + N in poplar plants.

Protein Accession	Position	CdN/Ck Ratio	Regulated Type	Cd N/Ck *p* Value	Amino Acid	Protein Description
A0A2K1WUE2	493	1.28	Up	0.0064	S	eukaryotic translation initiation factor isoform (eIF)
A0A2K1XQU0	1237	1.669	Up	0.0414	S	eukaryotic translation initiation factor 4G-like (eIF)
B9GP88	987	1.704	Up	0.018	T	eukaryotic translation initiation factor 4G isoform X1 (eIF)
B9GP88	988	1.899	Up	0.0271	S	eukaryotic translation initiation factor 4G isoform X1 (eIF)
A0A2K1XBQ3	26	1.458	Up	0.0485	S	ABC transporter G family member (ABC transporter protein)
A0A2K2ACX1	110	1.241	Up	0.00616	S	ABC transporter G family member (ABC transporter protein)
A0A2K1XB30	251	1.671	Up	0.028	S	zinc finger family protein (ZFP)
A0A2K1XB30	382	1.867	Up	0.0199	S	zinc finger family protein (ZFP)
A0A2K2ATR0	296	1.991	Up	0.00726	S	zinc-finger homeodomain protein 9-like (ZFP)
B9HN74	557	1.421	Up	0.0354	S	heat shock protein 70 (HSP70)
B9HV59	10	1.345	Up	0.0299	S	heat shock protein 70 (HSP70)
B9HV59	12	3.554	Up	0.00632	S	heat shock protein 70 (HSP70)
B9HV59	14	1.493	Up	0.0000634	T	heat shock protein 70 (HSP70)
U7E2Z8	604	2.388	Up	0.0185	S	heat shock-related family protein (HSP70)
W8PVS7	250	2.7	Up	0.0191	T	Peroxidase (POD)
A0A2K2C6G4	246	1.816	Up	0.0231	T	splicing factor 3B subunit 1-like (SF3B1)
A0A2K2C6G4	185	2.175	Up	0.0149	T	splicing factor 3B subunit 1-like (SF3B1)
A0A2K2C6G4	207	2.355	Up	0.0341	T	splicing factor 3B subunit 1-like (SF3B1)
A0A2K2C6G4	209	2.086	Up	0.0351	T	splicing factor 3B subunit 1-like (SF3B1)
A0A2K2C6G4	353	1.529	Up	0.0387	T	splicing factor 3B subunit 1-like (SF3B1)
A0A2K2C6G4	244	1.92	Up	0.0127	T	splicing factor 3B subunit 1-like (SF3B1)
A0A2K2C6G4	219	1.954	Up	0.0089	T	splicing factor 3B subunit 1-like (SF3B1)
A0A2K2C6G4	125	1.484	Up	0.0462	T	splicing factor 3B subunit 1-like (SF3B1)

## Abbreviations

HM	Heavy metal
N	nitrogen
Cd	cadmium
HSP70	heat shock protein 70
POD	peroxidase
ROS	reactive oxygen species
O_2_^−^	superoxide radicals
OH	hydroxyl radicals
H_2_O_2_	hydrogen peroxide
GSH	glutathione
PCs	phytochelatins
Chl a	chlorophyll a
Chl b	chlorophyll b
Pn_max_	the net rate of photosynthesis
R	transpiration rate
AQE	maximum quantum efficiency
g_s_	stomatal conductance
F_v_/F_m_	maximum quantum efficiency of PS II
ZFP	zinc finger protein
elF	eukaryotic translation initiation factor
SF3BI	eukaryotic translation initiation factor
GO	Gene Ontology
MAPK	mitogen activated protein kinase protein
PA2G4	proliferation-associated proteins 2G4
TCTP	translationally controlled tumor proteins
Prx	peroxiredoxin
GST	glutathione transferase
ABC transporter	the ATP-binding cassette transporter
TFs	transcription factors
Bhlh	helix-loop-helix
VPS	vacuolar protein sorting-associated protein
